# Carotid Free-Floating Thrombus Treated With a Combined Technique Using Embotrap III for Distal Protection

**DOI:** 10.7759/cureus.53775

**Published:** 2024-02-07

**Authors:** Yoichiro Nagao, Yuichiro Inatomi, Masaki Naganuma, Toshiro Yonehara, Makoto Nakajima

**Affiliations:** 1 Neurology, Saiseikai Kumamoto Hospital, Kumamoto, JPN; 2 Neurology, Graduate School of Medical Sciences, Kumamoto University, Kumamoto, JPN

**Keywords:** free-floating thrombus, acute ischemic stroke (ais), carotid stent, endovascular technique, stroke

## Abstract

Carotid free-floating thrombus (FFT) is a rare condition in patients with acute ischemic stroke. Recently, endovascular therapy for carotid FFT has been increasingly reported, but the strategy has not yet been established. We report a case of an acute stroke patient with a carotid FFT, who was successfully treated with a combination of the direct aspiration first-pass technique (ADAPT) and the Embotrap III (Cerenovus, Irvine, CA), specifically designed to prevent distal embolization. We propose the utility of distal embolic protection with Embotrap III for the treatment of patients with carotid FFT. A 71-year-old man who presented with sudden left hemiparesis was admitted to our hospital. Ultrasonography on admission revealed severe stenosis and an FFT at the origin of the right internal carotid artery. Thrombectomy with an aspiration catheter, accompanied by a stent retriever with distal basket Embotrap III for distal protection, was performed. After the FFT was safely aspirated, a carotid Wallstent (Boston Scientific, Marlborough, MA) was deployed in the stenosis. Follow-up ultrasonography showed neither FFT nor in-stent protrusion. The patient did not experience recurrence, as per clinical or radiological findings, and was discharged on day 11 without any neurological deficits. Embotrap III may be useful for a patient with a carotid FFT as distal protection during mechanical thrombectomies.

## Introduction

Carotid free-floating thrombus (FFT) is a rare condition with an incidence of 1.53% in patients with acute ischemic stroke [1]. Patients with FFT are at risk for worsening symptoms caused by distal embolization of the thrombus. The risk of transient ischemic attack, silent brain ischemia, and any stroke or death at 30 days was reported to be 17.1% in acute ischemic stroke patients with FFT [1].

Therapeutic strategies for acute stroke patients with carotid FFT have not yet been established. Recently, endovascular therapy for carotid FFT has been increasingly reported, in addition to conventional medical or surgical treatments. In most endovascular treatment cases, a filter wire is used for distal protection [2-4]. However, if a large FFT is caught in a filter, it is difficult to remove the filter owing to the risk of dissection.

We report a procedure using a combination of the direct aspiration first-pass technique (ADAPT) and Embotrap III (Cerenovus, Irvine, CA), which is a stent retriever used for protecting distal embolization. There have been no reported cases of utilizing Embotrap III or other stent retrievers for distal protection in FFT patients. We propose the utility of distal embolic protection with Embotrap III for the treatment of patients with carotid FFT.

## Case presentation

A 71-year-old man with hypertension was admitted to our hospital 8.5 hours after a sudden onset of left arm weakness. Physical examination on admission revealed a blood pressure of 192/95 mmHg and a heart rate of 68 beats/min. The patient's Glasgow Coma Scale score was 15 (E4V5M6). He had moderate dysarthria and left hemiparesis; his National Institutes of Health Stroke Scale (NIHSS) score was 8 points. Blood examination results were normal. Electrocardiography revealed a sinus rhythm.

**Figure 1:**
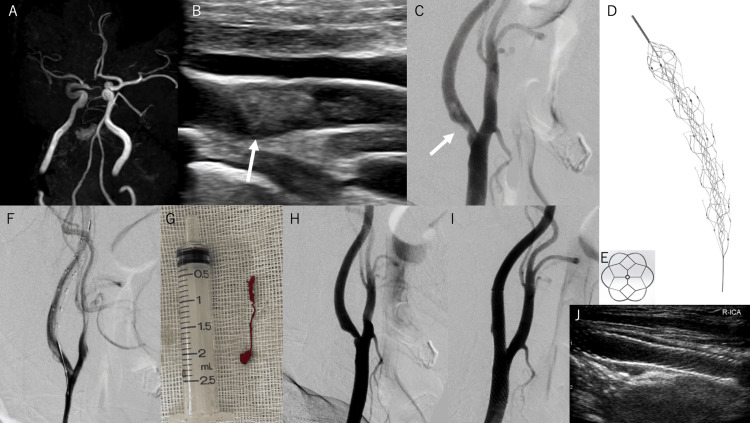
Treatment course of carotid artery occlusion A: The right distal ICA and middle cerebral artery cannot be visualized clearly on MRA. B: Longitudinal view of the right common carotid ultrasonography. An ulcerated plaque with FFT (white arrow), which moves synchronously with the heartbeat, is present beside the carotid plaque. C, F, H, I: Serial images from digital subtraction angiography. Carotid artery stenosis and FFT (C). Embotrap III (5×37 mm) was deployed in the distal ICA (F). After aspiration, the FFT was removed (H). After Wallstent deployment, the carotid lumen was dilated to a normal diameter (I). White arrows indicate FFT. D: Embotrap III. E: Distal basket of Embotrap III (front view). G: A string-like thrombus collected by aspiration. J: Follow-up carotid ultrasonography on day 1. Stent stenosis was not observed ICA: internal carotid artery; MRA: magnetic resonance angiography; FFT: free-floating thrombus

Diffusion-weighted images revealed a high-intensity lesion in the right frontal lobe. The Alberta Stroke Program Early CT score of the diffusion-weighted images was 9/10. On magnetic resonance angiography, the distal segment of the right internal carotid artery (ICA) and the middle cerebral artery could not be clearly visualized (Figure 1A). 

On carotid ultrasonography, carotid FFT (10.7 mm; Figure 1B) was observed at the origin of the right ICA. Digital subtraction angiography (DSA) revealed stenosis at the origin of the ICA and a filling defect suggesting FFT in the stenotic lumen of the ICA (Figure 1C).

Thrombolysis was not initiated due to the presentation occurring beyond the 4.5-hour window from symptom onset. After the administration of 200 mg aspirin and 300 mg clopidogrel, thrombectomy and carotid artery stenting were performed. Initially, a 9-Fr Optimo balloon guide catheter (Tokai Medical Products, Aichi, Japan) was navigated to the right common carotid artery. After inflating the balloon, a microcatheter was navigated to the distal ICA while aspirating from Optimo. Embotrap III (5×37 mm; Figure 1D, 1E) was deployed in the distal ICA (Figure 1F). After removing the microcatheter, the Embovac (Cerenovus, Irvine, CA) was navigated through the guidewire. Thereafter, the thrombus was aspirated using an Embovac at its proximal side. After aspiration, the Embovac was removed slowly with suction. A string-like thrombus was attached to the tip of the Embovac (Figure 1G). Subsequently, filling defects were not observed on DSA (Figure 1H) or intraoperative carotid ultrasonography. After inflating the 9-Fr Optimo balloon, we performed aspiration through it while removing the Embotrap III and found a small thrombus attached to the distal tip of the Embotrap III. There was no thrombus observed in the ICA lumen.

A carotid Wallstent (10×24 mm; Boston Scientific, Marlborough, MA) was deployed at the stenotic lesion, and post dilatation was performed using a 5×20 mm Sterling (Boston Scientific, Marlborough, MA). The carotid lumen was dilated to a normal diameter (Figure 1I), and no occlusion of the distal artery was observed.

Pathologically, the FFT was a fibrin-rich thrombus composed mainly of fresh red blood cells and fibrin. Bacterial bodies, cholesteric cleft, and fibrous tissue suggesting the carotid web were not detected.

Aspirin 200 mg/day, clopidogrel 75 mg/day, argatroban 60 mg/day, and edaravone 60 mg/day were started on day 1. The next day, his left arm weakness fully recovered, and post-treatment carotid ultrasonography showed no in-stent stenosis or FFT on day 1 (Figure 1J). No abnormalities were detected on transthoracic echocardiography or 24-hour Holter electrocardiography. The patient was diagnosed with atherothrombotic stroke caused by carotid FFT, and dual antiplatelet therapy (aspirin 100 mg/day and clopidogrel 75 mg/day) was continued for three months. Postoperative MRI on day 4 showed no new ischemic lesions. The patient was discharged on day 10 with mild sensory disturbance in the left upper arm and an NIHSS score of 1. He had no neurological deficits, and restenosis of the lesion was not observed on carotid ultrasonography three months after onset.

The patient's consent for submission of this paper has been obtained.

## Discussion

In the present case, we focused on the distal basket of Embotrap III, which was devised for thrombus retrieval and the prevention of distal embolization. To our knowledge, this is the first report that uses Embotrap III for distal protection in a patient with carotid FFT. Although the thrombus was retrieved only by an aspiration catheter in terms of results, the device was considered to be well qualified for distal protection in these cases, with no need for complicated procedures.

However, a therapeutic strategy for acute stroke with carotid FFT has not yet been established. Distal embolization can occur during anticoagulation therapy [5]; additionally, surgery is considered unsafe due to invasive general anesthesia or distal thrombus [6].

Recently, in addition to conventional treatments, endovascular thrombectomy for carotid FFT has been reported [2,4,5,7-14]. However, when balloon protection is used for distal protection, complete interruption of the ICA flow is undesirable in patients without hemodynamic intolerance. It may not be possible to safely re-sheathe a filter wire because of the risk of vasospasm or dissection [15]. If a large FFT is caught in the filter, it is difficult to remove the filter wire itself [16]. There have been no reported cases of utilizing Embotrap III or other stent retrievers for distal protection in FFT patients.

The Embotrap III used in our case (Figure 1D) has a distal closed structure at the tip of the stent that differentiates it from other stent retrievers. This unique design enables it to capture thrombi with a distal basket (Figure 1E). And the Embotrap III can be retrieved with a low risk of vessel damage because it is very soft. Moreover, Embotrap III can be used in combination with clot retrieval and thrombus aspiration. Therefore, we consider that this technique of using Embotrap III as distal protection can be the first choice for the treatment of FFT.

However, because the Embotrap III basket is rough, embolization by small thrombi may occur. Therefore, a balloon protection device at the proximal portion, such as the Optimo balloon guide catheter, should be used concomitantly. Including comparisons between stent retrievers in randomized controlled trials, validation of the use of stent retrievers for protection requires the accumulation of cases.

## Conclusions

This is a case of carotid FFT, in which the Embotrap Ⅲ was used not only for thrombectomy but also for distal embolism prevention. In the present case, successful recanalization was achieved, with no new ischemic lesions. The Embotrap Ⅲ may be considered as the distal protection for thrombectomy in cases of carotid FFT.
